# Multi-family therapy for veteran and refugee families: a Delphi study

**DOI:** 10.1186/s40779-018-0170-9

**Published:** 2018-08-06

**Authors:** Elisa van Ee

**Affiliations:** 1 Psychotraumacentrum Zuid Nederland, Reinier van Arkel, Bethaniestraat 10, 5211 LJ ‘s-Hertogenbosch, the Netherlands; 2 Behavioural Science Institute, Radboud University, Montessorilaan 3, 6525 HR Nijmegen, the Netherlands

**Keywords:** PTSD, Veteran, Refugee, Multi-family therapy, Parenting, Expert opinion

## Abstract

**Background:**

Research indicates that Posttraumatic stress disorder (PTSD) has an extensive impact on family relationships. Nevertheless, there is a dearth of empirically supported interventions addressing family functioning and PTSD. In the Netherlands, it is considered good clinical practice to offer multi-family therapy (MFT) to veteran and refugee families. MFT for traumatized families aims to address the dysfunctional family patterns that have evolved to address the consequences with trauma.

**Method:**

The aim of this study is to generate a common framework for the practical impact and active ingredients of MFT in families confronted with trauma. The Delphi method was used to study the expert opinion of 11 therapists in Dutch expert trauma institutes.

**Results:**

The results indicate that MFT is a promising treatment for families dealing with the consequences of trauma. According to experts, positive outcomes include an increased understanding between family members, particularly visible in the de-escalation of conflicts within the family, and improved parenting. One explanation for the effectiveness of MFT with these target groups is its defining feature of therapy with several families.

**Conclusions:**

The findings support the importance of considering family relationships and the family context in interventions for traumatized individuals.

## Background

There is a conceptual gap between the concept of posttraumatic stress disorder (PTSD), which defines a traumatized individual who experiences distress, suffering and impairment, and the significant distress, suffering and impairment of families as the result of PTSD of one family member. A review of traumatized parents and relational patterns with their children showed consistent negative associations between increased parental symptoms of PTSD, parent functioning, a reduced quality of the parent-child relationship and child functioning [1, 2]. A prospective longitudinal study of a population at risk established a relationship between maternal PTSD and insecure, particularly disorganized, child attachment [3]. Furthermore, a meta-analysis of the association between parents’ PTSD and children’s psychological distress revealed that both paternal and maternal PTSD symptoms were significantly associated with child distress [4]. In addition, in traumatized populations, contextual variables such as work-related stress, finances, relationship difficulties and lack of social support have been associated with parenting and child wellbeing [5–7].

A review of the relational patterns between caregivers with PTSD and their children showed that traumatization can cause parenting limitations, and these limitations can disrupt the development of the child. To understand the complex relational patterns, many factors need to be considered (e.g., parental symptoms of PTSD, co-morbidity in parental psychopathology, and childhood trauma of the parent). Mechanisms such as mentalization (the capacity to perceive and understand mental states of the self and the child that help to explain and predict feelings, thoughts and behavior), attachment, and physiological factors offer a valuable perspective; however, to understand the impact of parental traumatization on children, the need for a transactional perspective, the inclusion of child factors, is essential [1]. These findings support the importance of considering family relationships and the family context in interventions for traumatized individuals. Despite the indications of an extensive impact of PTSD on the family, particularly children, there is a dearth of empirically supported interventions addressing family functioning and PTSD [1, 8, 9].

In the Netherlands, it is considered good clinical practice to offer multi-family therapy (MFT) to veteran and refugee families, as the experience of trauma and violence leads to changes in multiple dimensions of individual and family functioning, and systemic approaches, such as MFT, sensitize the social and cultural context in which the meanings of individual and family functioning are shaped [10–12]. MFT can be defined as a deliberate psychosocial intervention with two or more families and at least two generations in the family. Sessions focus on problems or concerns shared by all families in attendance [13]. MFT aims to elicit behavioral changes in family members through the restructuring of interactional patterns in families. The interactions and processes in different subsystems facilitate change in individuals and families, as different perspectives and opportunities to experiment with new behavior are generated [14]. MFT for veteran and refugee families aims to address the dysfunctional family patterns that have evolved to deal with, as a family, the consequences of traumatization. Veteran and refugee families participate in separate MFT groups, but these families share their past belonging to a certain group, their present isolation, the perception of trauma and the consequences of traumatization through the lens of a family system or group [15–17].

Dutch expert trauma centers dealing with veterans and refugees offer MFT. Nevertheless, evidence of the efficacy of MFT comes from studies in families dealing with severe depression, obsessive compulsive disorder, substance abuse, abuse and neglect, and eating disorders [14]. In contrast with these studies, MFT for veteran and refugee families is not directed at reducing the symptomatology of PTSD but rather is aimed at reducing the consequences of traumatization by improving functioning and is therefore characterized by a variability in change mechanisms and treatment outcomes. Thus, the efficacy and effectiveness of MFT for the treatment of the consequences of PTSD on families have not yet been systematically studied.

Complex interventions are conventionally defined as interventions with several interacting components [18]. MFT fits this definition, as it has been designed to be tailored towards the needs of families from a variety of groups and with a variability of outcomes. These different components and the variability of outcomes add to the complexity of evaluating the MFT intervention and establishing causal chains linking the intervention with the primary outcome(s).

The Medical Research Council proposes that the development and evaluation of these interventions require an understanding of its practical impact and whether it works in everyday practice, as well as a good theoretical understanding of how the intervention causes change, what the active ingredients are and how they exert their effect [18]. Only by addressing these questions is it possible to understand and evaluate the intervention and to design more effective interventions.

The Delphi method can be particularly useful to systematically gather expert knowledge and the understanding of an intervention and, in this case, to prioritize the change mechanisms and treatment outcomes [19, 20]. To initiate the development of a more comprehensive evidence base, the aim of this study is to generate a common framework of the practical effect and the active ingredients of MFT in families confronted with PTSD. This framework would result from the work with families from different backgrounds dealing with the chronic consequences of traumatization and could form a stepping stone for systematic studies of the efficacy and effectiveness of MFT in families confronted with PTSD.

## Method

The Delphi method [19] is well-suited to study a relatively small group of experts whose knowledge and opinions are a guide to best practice. It also helps to promote agreement among these experts. The Delphi methodology was developed by the RAND Corporation in the 1950s and is defined by four basic characteristics:

1. Repeated individual questioning of the experts
2. Anonymity
3. Controlled feedback
4. Prioritizing the information

The research question is formulated in a survey of open questions and is then sent to a sample of suitably qualified experts. The experts respond anonymously, and their responses are synthesized into a list that is fed back to the experts for their consideration. Issues, concepts and suggestions raised by experts are fed back to the group. The experts could adjust their response until consensus or another predetermined point in the process is reached [19].

All therapists currently using MFT with refugee and veteran families in Dutch expert trauma institutes were invited to participate (criterion sampling, *N* = 15). These centers offer specialized and highly specialized trauma care. Specialized units deliver care to refugees and veterans, as well as their families. Care is delivered to refugees from all over the world, for example, from Afghanistan, China, Eritrea, Iraq, Iran, Somalia, and Syria. Most of these refugees have experienced multiple traumatic experiences, some examples of which are imprisonment, being wounded, combat situations, rape, murder of a relative or friend, and torture. Care is delivered to veterans who experienced traumatic events during missions, for example, in Afghanistan, Cambodia, former Yugoslavia, Iraq, and Lebanon. Most veterans experience multiple traumatic experiences as well; examples are imprisonment, being wounded, combat situations, and death of a friend or colleague.

In the first round, a survey was performed among these experts to gain insight into therapeutic outcomes, change mechanisms and essential techniques. Experts were prompted to both generate knowledge on their perspective on MFT and ideas on the perspective of families using MFT. The experts were asked to generate an exhaustive list in response to the following questions:

1. Please indicate what you believe are the core positive therapeutic outcomes of MFT.
2. Please indicate what you believe are the change mechanisms that bring about these positive therapeutic outcomes of MFT.
3. Please indicate what you believe are the essential techniques for positive outcomes of MFT.
4. Please indicate what families believe are the core positive therapeutic outcomes of MFT.
5. Please indicate what you believe are the mechanisms that withhold positive outcomes of MFT.
6. Please indicate what you believe are the (potential) negative outcomes of participating in MFT for family members.
7. Please indicate what families believe are the (potential) negative outcomes of participating in MFT.
8. Please indicate why MFT can be used as an intervention to deal with problems associated with a traumatized parent.

Two coders categorized the responses of the experts into themes until a consensus was reached. Duplicates and redundancies were removed. The result of this analysis was a list of anonymous expert responses to each question. In the second round, this list was fed back to the experts who responded in the first round to determine prioritization. Experts were requested to score each response on a five-point Likert scale for the criteria: relevancy and frequency (1 = not relevant or not frequent, 5 = very relevant or very frequent). For example, each expert rated the relevancy of the increased understanding between family members and the frequency of the increased understanding between family members as a positive outcome of MFT. In addition, experts were given the opportunity to provide feedback on the generated list. In the third round, all participants in the second round were requested to respond to the anonymous feedback of individual experts. Consensus was defined as an agreement between experts of at least 75% on ‘relevancy’. The weight of an item was defined as the mean score on ‘frequency’ multiplied by the percentage of ‘relevancy’. The weight of the items is reported in this article.

## Results

Eleven experts participated in the first round, ten in the second and nine in the third round (response rate = 73.33%, attrition rate = 18.18%). Participants were psychiatrists, clinical psychologists, family therapists or psychiatric nurses, mostly female (80%), with a mean age of 51.7 years (S.D. = 9.14) and with an average of 5.10 years of work experience in MFT (S.D. = 1.60).

Table 1 shows the results for the core positive therapeutic outcomes of MFT according to the therapists and families, as rated by the experts. The experts give the highest weight to an increased understanding between family members, which was particularly visible in the de-escalation of conflicts within the family. Improved parenting was ranked the second. Examples given were reduced parentification (a process of role reversal), increased sensitivity to the children’s needs and increased self-esteem as a parent. Where the experts did not rank contact with other families as a positive outcome, according to these experts, the families themselves do. Of further importance to families, according to the experts, was overcoming isolation and mutual recognition, which was defined as feeling less like the only one with the problem.

**Table 1:** Positive therapeutic outcomes of MFT rated by experts

Experts’ opinion	Score	Families’ opinion (according to experts)	Score
Increased understanding, de-escalation of conflicts	4.10	Contact with families, overcoming isolation	4.60
Improved parenting	3.70	Mutual recognition	4.50
Increase in support of family members	3.60	Being aware and reflective	4.10
Secure bonding	3.50	Improved parenting	3.90
Changed patterns^a^	3.38	Higher self-esteem	3.80
Openness in communication	3.30	Support of professionals	3.04
Increased resilience	3.10	Coping skills and resources	2.64
Learning ways to regulate and show emotions	3.10	**–**	**–**

*MFT* Multi-family therapy; ^a^In dealing with (traumatic) stress and family life, replacing unhealthy with healthy patterns; − No data

Mechanisms either supporting or withholding positive changes are shown in Table 2. Working with other families is the key change mechanism of MFT where experts mention different components: observing and thinking about other parents and/or children, being able to reflect on others and use other families as a mirror to one’s own problems (subscore 4.44), and receiving advice and feedback from other peers (subscore 4.56), thereby using each other’s process to grow (subscore 3.56). Several of the mechanisms that withheld positive change were described as contrasts: either too much talking or too little action, too strong group dynamics or not enough group dynamics, and therapists that were either too inactive or too active or directive. Finally, addiction was mentioned as withholding change but is also a contra-indication for participation in MFT.

**Table 2:** Mechanisms in MFT rated by experts

Experts’ opinion	Score	Families’ opinion (according to experts)	Score
Working with other families	4.50	MFT without individual therapy	2.56
Recognition and identification	4.38	Too much talking, too little action	2.56
Mentalization	4.20	Group dynamics (too strong/weak)	2.52
Unraveling behavioral sequences	4.00	Therapist (too inactive/active)	2.32
Positive atmosphere, context for learning	3.80	Insufficient safety at home	2.25
Generating hope and multiple perspectives	3.78	Unclear rationale of therapy	2.00
Adults voice how they are affected	3.46	Lack of motivation, no-show	1.68
Ownership of the problem	3.44	Addiction	1.44
Children voice how they are affected	3.06	–	–

*MFT* Multi-family therapy; − No data

All potential negative outcomes, according to experts or families (rated by experts), were scored low in frequency but high in relevancy (Table 3). No treatment effect, either individually or as a family, was rated as not frequent, but all were very relevant (positions one and three). When group dynamics become too strong, collective thinking can develop that leads to demoralization and the sense that change is not possible (positions two and five).

**Table 3:** Potential negative outcomes of MFT rated by experts

Experts’ opinion	Score	Families’ opinion (according to experts)	Score
Insufficient decrease of symptoms	2.72	Disappointment	2.97
Groupthink: we cannot change	2.61	Treatment causes stress	2.48
Insufficient improvement	2.56	Unchanged psychopathology	2.44
Increase in unsafety at home	2.43	**–**	–
Demoralization	2.24	–	–

*MFT* Multi-family therapy; − No data

Experts reached a consensus over five reasons for applying MFT with families confronted with trauma. These reasons were considered as most present in and most relevant for clinical practice:

1. Others with similar problems tend to understand you easier (4.7)
2. Generating hope and multiple perspectives (4.44)
3. Sharing and connecting with other people (4.3)
4. Mentalization (3.8)
5. Families become experts in their own process (3.7)

## Discussion

The aim of this study was to generate a common framework for the practical effect and active ingredients of MFT in families confronted with PTSD. The results show that MFT with veteran or refugee families is clearly aimed at the treatment of the consequences of PTSD on a systemic level. MFT supports families that have lost sight of each other and the environment and who got stuck in a pattern that led to the loss of hope and perspective. On a systemic level, these consequences can be conceptually linked to the consequences of complex traumatization: loss of trust in people, loss of meaning, loss of control, loss of the ability to mentalize, and loss of a future perspective. Trauma to individuals has the potential to reverberate throughout the family system.

MFT is a promising treatment for veteran and refugee families dealing with the consequences of trauma. One explanation for the reported effectiveness of MFT with these target groups is its defining feature of therapy with several families. Both veterans and refugees can be particularly prone to experiencing the importance of being part of a group or the loss of being part of a group. In contrast with experts who value contact with other families as a vehicle toward change, the veteran and refugee families value this as being the most significant positive outcome of MFT. Even though belonging to a group and groupthink can become too strong and counterproductive, it is exactly this feature of belonging that can create a window of opportunity for difficult-to-reach populations, such as veterans and refugees, to open up to interventions on a systemic level.

MFT was designed as a generic systemic intervention for complex family problems. Specific to using MFT with families confronted with trauma is that MFT should not be a stand-alone treatment but instead should be combined with individual trauma-focused therapy. These results are in line with a study on the efficacy of recommended treatments for veterans with PTSD, which found that group therapy alone was not effective, whereas a combination of individual trauma-focused therapy and group therapy had the highest combined effect size [21]. A combination of MFT with individual therapy aimed at the symptoms of PTSD is necessary to treat those symptoms, as well as to sustain long-term changes on a systemic level.

Despite being promising, MFT for families confronted with trauma is also in need of refinement. Due to its generic design, MFT protocols are lacking, and it is in the hand of the therapist to design a completion of MFT for the specific target group in the specific context of an institute. Considering the mechanisms that withhold positive change, the effectiveness of MFT for families confronted with trauma can be improved substantially with clear guidelines for the intervention. Active ingredients, such as working with other families, recognition and identification, mentalization and unraveling behavioral sequences, should be core treatment components in such guidelines. Research into the efficacy and effectiveness of MFT can benefit from clear guidelines as well.

A strength of this study lies in the evaluation of a complex intervention using the Delphi method. Until now, no systemic studies into MFT for families confronted with PTSD are available. The generalizability of the results is, however, a limitation. This study was performed with Dutch experts only working in Dutch trauma expert institutes and using MFT with veterans and refugees. In addition, we did not ask the opinion of families directly but gathered this information via the experts. Future studies could qualitatively study the perspective of families and quantitatively focus on the effect of MFT on family functioning and more specific communication, conflict, parenting, bonding and emotion regulation, as these concepts are primary outcomes according to the experts.

## Conclusions

The aim of this study was to generate a common framework for the practical effect and active ingredients of MFT in families confronted with PTSD. The results show that MFT with veteran or refugee families is clearly aimed at the treatment of the consequences of PTSD on a systemic level and is a promising treatment for families dealing with the consequences of trauma. Increased understanding between family members, which was particularly visible in the de-escalation of conflicts within the family, and improved parenting are rated by experts as the most important outcomes. One explanation for the reported effectiveness of MFT with these target groups is its defining feature of therapy with several families. The findings support the importance of considering family relationships and the family context in interventions for traumatized individuals and could form a stepping stone for systematic studies into the efficacy and effectiveness of MFT in families confronted with PTSD.

## Abbreviations

MFT: Multi-family therapy
PTSD: Posttraumatic stress disorder
